# Improvement of Image Quality and Diagnostic Performance by an Innovative Motion-Correction Algorithm for Prospectively ECG Triggered Coronary CT Angiography

**DOI:** 10.1371/journal.pone.0142796

**Published:** 2015-11-16

**Authors:** Zhen-Nan Li, Wei-Hua Yin, Bin Lu, Hong-Bing Yan, Chao-Wei Mu, Yang Gao, Zhi-Hui Hou, Zhi-Qiang Wang, Kun Liu, Ashley H. Parinella, Jonathon A. Leipsic

**Affiliations:** 1 Department of Radiology, State Key Laboratory of Cardiovascular Disease, Fuwai Hospital, National Center for Cardiovascular Diseases, Chinese Academy of Medical Sciences and Peking Union Medical College, Beijing, People’s Republic of China; 2 Department of Cardiology, State Key Laboratory of Cardiovascular Disease, Fuwai Hospital, National Center for Cardiovascular Diseases, Chinese Academy of Medical Sciences and Peking Union Medical College, Beijing, People’s Republic of China; 3 Department of Radiology, College of Medicine, Medical University of South Carolina, Charleston, South Carolina, United States of America; 4 Department of Radiology and Division of Cardiology, University of British Columbia, Vancouver, BC, Canada; University of Washington School of Medicine, UNITED STATES

## Abstract

**Objective:**

To investigate the effect of a novel motion-correction algorithm (Snap-short Freeze, SSF) on image quality and diagnostic accuracy in patients undergoing prospectively ECG-triggered CCTA without administering rate-lowering medications.

**Materials and Methods:**

Forty-six consecutive patients suspected of CAD prospectively underwent CCTA using prospective ECG-triggering without rate control and invasive coronary angiography (ICA). Image quality, interpretability, and diagnostic performance of SSF were compared with conventional multisegment reconstruction without SSF, using ICA as the reference standard.

**Results:**

All subjects (35 men, 57.6 ± 8.9 years) successfully underwent ICA and CCTA. Mean heart rate was 68.8±8.4 (range: 50–88 beats/min) beats/min without rate controlling medications during CT scanning. Overall median image quality score (graded 1–4) was significantly increased from 3.0 to 4.0 by the new algorithm in comparison to conventional reconstruction. Overall interpretability was significantly improved, with a significant reduction in the number of non-diagnostic segments (690 of 694, 99.4% vs 659 of 694, 94.9%; P<0.001). However, only the right coronary artery (RCA) showed a statistically significant difference (45 of 46, 97.8% vs 35 of 46, 76.1%; *P* = 0.004) on a per-vessel basis in this regard. Diagnostic accuracy for detecting ≥50% stenosis was improved using the motion-correction algorithm on per-vessel [96.2% (177/184) vs 87.0% (160/184); *P* = 0.002] and per-segment [96.1% (667/694) vs 86.6% (601/694); *P* <0.001] levels, but there was not a statistically significant improvement on a per-patient level [97.8 (45/46) vs 89.1 (41/46); *P* = 0.203]. By artery analysis, diagnostic accuracy was improved only for the RCA [97.8% (45/46) vs 78.3% (36/46); *P* = 0.007].

**Conclusion:**

The intracycle motion correction algorithm significantly improved image quality and diagnostic interpretability in patients undergoing CCTA with prospective ECG triggering and no rate control.

## Introduction

Coronary computed tomography angiography (CCTA) is widely used as a noninvasive imaging tool for identification and exclusion of obstructive coronary artery disease (CAD). Diagnostic accuracy improved as the technology evolved from 16- to 64- to 320-slice machines, along with an increase in temporal and spatial resolution and a decrease in the number of non-assessable coronary segments [1,2]. In particular, the impressively high negative predictive value (NPV), both at the patient and vessel level, establishes CCTA as an effective alternative to invasive coronary angiography (ICA) for ruling out CAD [2,3]. Improved CT technology has also been developed to reduce radiation dose. For example, prospective electrocardiogram (ECG) triggering protocols are used as an alternative to the retrospective ECG gating acquisition and iterative reconstruction algorithms are increasingly replacing the filtered back projection, the traditional method of image reconstruction [4,5]. Prospective ECG-triggered low-dose CCTA is increasingly used in clinical routine.

However, factors such as elevated heart rate, arrhythmia, high body mass index, and high coronary calcium levels make CCTA prone to image artifacts, especially motion artifacts. The use of rate-lowering medications before CCTA is recommended in the current clinical guidance documents to prevent motion artifacts [6]. Due to adverse influence of motion artifacts on image quality and diagnostic performance of CCTA, various effective means have been applied to alleviate motion artifacts, including use of dual-source CT with dual x-ray sources and detector arrays, high-pitch helical image acquisition, and image reconstruction during the appropriate segment of the cardiac cycle. However, no techniques have been developed to reduce motion artifacts in regard to rate control [3,7]. Recently, a new image reconstruction technology applied to CCTA obtains information from a minimum of 2 cardiac phases within a single cardiac cycle, which compensates for coronary motion both by prospective and retrospective ECG gating [8]. This innovative motion-correction approach may permit a relatively high heart rate for CCTA while preserving image quality and diagnostic performance, especially for prospectively ECG-triggered low-dose CCTA, which requires a low heart rate.

Previous studies have shown that the new motion-correction algorithm can improve image quality, interpretability and diagnostic accuracy of CCTA despite insufficient or absent rate-control medications, which may offer guidance for clinical practice [8–15]. Nevertheless, these studies either were performed with retrospective ECG gating, which generates relatively high radiation dose, or performed with prospective ECG triggering without using the ICA as the reference standard. More evidence is needed on the effect of this new algorithm in a clinical setting utilizing low-dose CCTA with ICA as the reference standard. Therefore, we undertook this prospective study to evaluate the effect of the motion compensation reconstruction technique on image quality, interpretability, and diagnostic performance in patients undergoing prospective ECG-triggered CCTA without rate control using the ICA as reference standard.

## Materials and Methods

### Study subjects

**Fig 1:**
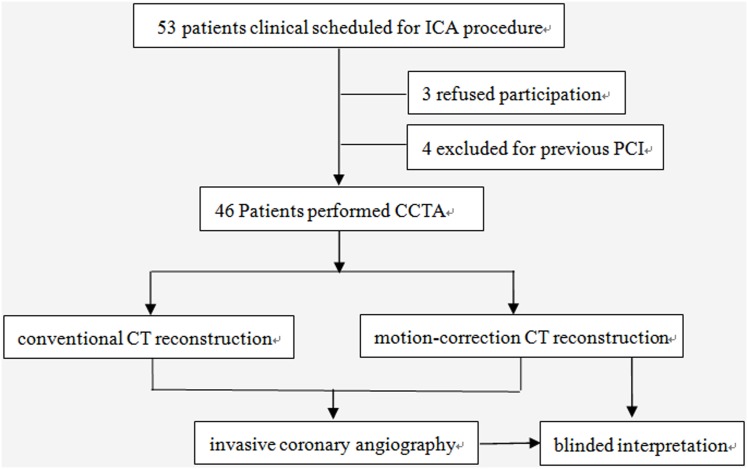
Study flowchart of enrollment.

This single-center prospective study complied with the Declaration of Helsinki and was approved by the Institutional Review Board of Fuwai hospital. All patients signed informed consents. A total of 53 patients were consecutively approached for participation in our study. We Included subjects who were (1) of age between 18 and 80 years; (2) highly suspected of having obstructive coronary artery disease and were accordingly referred for ICA; (3) in agreement to perform investigational CCTA (provided informed consent) before their clinical scheduled ICA. We excluded: (1) history of prior percutaneous coronary intervention (PCI) or coronary artery bypass graft; (2) clinical instability such as acute coronary syndrome and severe congestive heart failure; (3) impaired renal function (serum creatinine >120 umol/l); (4) prior anaphylactic allergy to iodinated contrast materials. Patients receiving rate-lowering medications would be asked to discontinue these medications for at least 24 hours before investigational CCTA. Of the 53 patients, 3 refused participation and 4 were excluded for previous PCI. Finally, 46 patients were enrolled in our study. Study flowchart of enrollment was shown in Fig 1.

### CCTA scanning protocols

One or 2 days before their clinical scheduled ICA, all subjects underwent contrast-enhanced CCTA on a Discovery CT750 HD scanner (GE Healthcare, GE, USA). Acquisition parameters included 64×0.625 mm detector collimation and 350 ms gantry rotation time. All studies were performed in a craniocaudal direction with prospective gating during breath inspiration. The scan range extended from the level of the carina to just below the dome of the diaphragm. 100 kV tube potential was used for patients with a body mass index (BMI) < 30 kg/m^2^, 120kV for BMIs > 30 kg/m^2^, and 80kV for BMIs < 20 kg/m^2^. X-ray tube currents were adjusted individually for each patient, depending on BMI. For contrast medium enhancement, automated bolus tracking was used in a region of interest within the ascending aorta, with a signal attenuation trigger threshold of 150 Hounsfield units (HU) and a 6-s scan delay. We used a triple-phase contrast medium injection protocol, which consisted of 50 to 60 ml of undiluted contrast agent (iopromide [Ultravist] 370 mgI/ml, Bayer Healthcare, Berlin, Germany) followed by a 30-ml 30%:70% mixture of contrast medium and saline, and a 40-ml saline chaser bolus, all injected with flow rates of 4 to 5 ml/s. All prospective ECG-triggered scans were performed with relatively small X-ray window: center of the image window set at 75% of the R-R cycle (padding was 80 ms) for patients with heart rate < 65 beats/min; center at 60% of the R-R cycle (padding was 180 ms) for patients with heart rate 65–75 beats/min; center at 45% of the R-R cycle (padding was 90 ms) for patients with heart rate > 75 beats/min.

### Image reconstruction

All CT scans were reconstructed using both the standard reconstruction method that is generated for 75% and 45% of the R-R interval as part of the clinical routine as well as the new motion-correction reconstruction technique (Snap Shot Freeze; GE Healthcare). The motion-correction reconstruction technique obtains information from adjacent cardiac phases within a single cardiac cycle to detect vessel path and velocity in order to determine the actual vessel position at the prescribed target phase. This technique compensates for any residual motion at that phase, effectively compressing the reconstruction temporal window. As previously described, this approach works on a per-vessel and per-segment basis to correct for differing degrees of motion for each voxel of the coronary vessel. Unlike conventional reconstruction techniques, this technique directly targets coronary-specific motion by adaptively compressing the temporal window within affected, localized regions. Because this method characterizes motion within a single cardiac cycle, it is less susceptible to beat-to-beat inconsistencies as well as heart or gantry period resonance points that can limit multisegment reconstruction [8].

### Image quality analysis

Data sets of each CCTA examination were transferred to a picture archiving and communication systems (PACS) diagnostic work station (Advantage Windows, GE Healthcare, Waukesha, Wisconsin). Two observers (with 13 and 10 years of experience in cardiovascular imaging, respectively), who were blinded to the reconstruction technique and unaware of ICA results, independently evaluated all CCTA studies in random order. Conventional standard reconstruction and motion-correction reconstruction image series of the same patient were presented a minimum of 4 weeks apart in order to minimize the potential reader recall bias. Both observers used transverse sections and standardized window settings (window level 300 HU, width 800 HU) to assess image quality. Image quality was graded on a per-segment level using a 4-point scoring system: score of 1, poor, nondiagnostic; 2, moderate, artifacts, but diagnosis still possible; 3, good, minor artifacts; and 4,excellent, no artifact; Image quality was also assessed on a per-vessel level, including the left main, left anterior descending, left circumflex, and right coronary arteries. Interpretability was defined as a score >1 on per-segment level, and was also evaluated on per-vessel and per-patient levels. If one segment was defined as not interpretable, the corresponding coronary artery and patient were also rated as not interpretable. A final consensus read was performed to resolve discrepancies in interpretation between the 2 observers regarding image quality scores and interpretability.

### Diagnostic performance analysis

For evaluation of coronary artery luminal integrity, the observers used transverse sections as well as secondary visualization methods provided by the interpretation platform, including maximum intensity projections, curved multiplanar reformats, and 3-dimensional volume-rendered technique. The extent of luminal stenosis of all evaluable segments was graded as a percentage of the vessel diameter by the CCTA readers using a semiquantitative scale. Stenosis severity was assigned a semiquantitative score: 0, 0% luminal stenosis; 1, 1%-49% luminal stenosis; 2, 50%-69% luminal stenosis; 3, 70%-99% luminal stenosis; 4, 100% luminal stenosis. The presence of luminal stenosis was assessed on a per-segment, per-vessel, and per-patient level, using the 18-segment model according to the Society of Cardiovascular Computed Tomography Guidelines [6]. A third observer adjudicated the score in cases of disagreement. Diagnostic performance characteristics including diagnostic accuracy, sensitivity, specificity, positive predictive value (PPV), negative predictive value (NPV) for detecting ≥50% stenosis were compared between the motion-correction reconstruction and conventional reconstruction group on per-patient, per-vessel, and per-segment levels using ICA as reference standard. If one segment was defined as ≥50% stenosis, the corresponding coronary artery and patient were also deemed as having ≥50% stenosis. Coronary segments and the corresponding coronary arteries and patients not able to be interpreted due to motion artifacts or severe calcification were classified as positive for diagnostic performance analysis.

### ICA performance

Selective ICA was performed according to standard technique, using 5-F or 6-F diagnostic catheters. A minimum of 5 projections for the left and 2 projections for the right coronary artery were obtained. Quantitative coronary angiography analysis was performed by 2 experienced cardiologists blinded to CCTA results, the severity of coronary stenosis was quantitatively evaluated, ≥50% luminal narrowing of the coronary artery diameter was judged as having significant stenosis.

### Radiation dosimetry

The dose-length product (DLP) for all CCTA acquisitions was recorded from the automatically generated patient dose report. Effective radiation dose was calculated as the product of DLP multiplied by a conversion coefficient for chest (k = 0.014 mSv [mGy cm]-1). Radiation dose from ICA was not estimated in the conduct of this study.

### Statistical analysis

Statistical analysis was performed with SPSS version 16.0 (SPSS, Chicago, Illinois). Continuous variables were expressed as mean±SD, and categorical variables as absolute numbers and percentages. Ordinal variables were summarized as medians and 25th and 75th percentiles. Normally distributed variables were compared using the Student t test, non-normally distributed variables using the Wilcoxon test. The χ2 test or Fisher exact test, as appropriate, were used for categorical variables. Inter-reader agreement between the 2 observers for the assessment of image quality and coronary stenosis severity at their initial interpretation before reconciliation was determined using Cohen,s Kappa coefficient. Levels of agreement on values were defined as follows: k = 0.81 to 1.00, excellent; k = 0.61 to 0.80, good; k = 0.41 to 0.60, moderate; k = 0.20 to 0.40, fair; k < 0.20, poor. For each group, diagnostic characteristics were calculated and compared with those of ICA, with a cutoff of coronary artery stenosis of 50% or greater. Receiver-operating characteristic (ROC) analyses were performed to compare diagnostic performance. p < 0.05 was considered statistically significant.

## Results

### Patients characteristics

The mean age was 57.6±8.9 years, (range: 40–77 years), 35 patients (76.1%) were male. Mean heart rate during scan was 68.8±8.4 beats/min (range: 50–88 beats/min) without rate control medications. The dose-length product and effective radiation dose was 238±46.4 mGycm and 3.3±0.6 mSv respectively. Other clinical characteristics are given in Table 1.

**Table 1:** Patients characteristics.

Patient characteristics	(n = 46)
**Age, yrs**	**57.6±8.9**
**Male**	**35 (76.1)**
**Body mass index, Kg/m** ^**2**^	**25.8±4.1**
**Heart rate, beats/min**	**68.8±8.4**
**Hypertension**	**30 (65.2)**
**Dyslipidemia**	**25 (54.3)**
**Smoking**	**29 (63)**
**Diabetes mellitus**	**14 (30.4)**
**Family history of CAD**	**17 (37)**

Values are mean ±SD or n (%) CAD = coronary artery disease

### Image quality and interpretability

**Fig 2:**
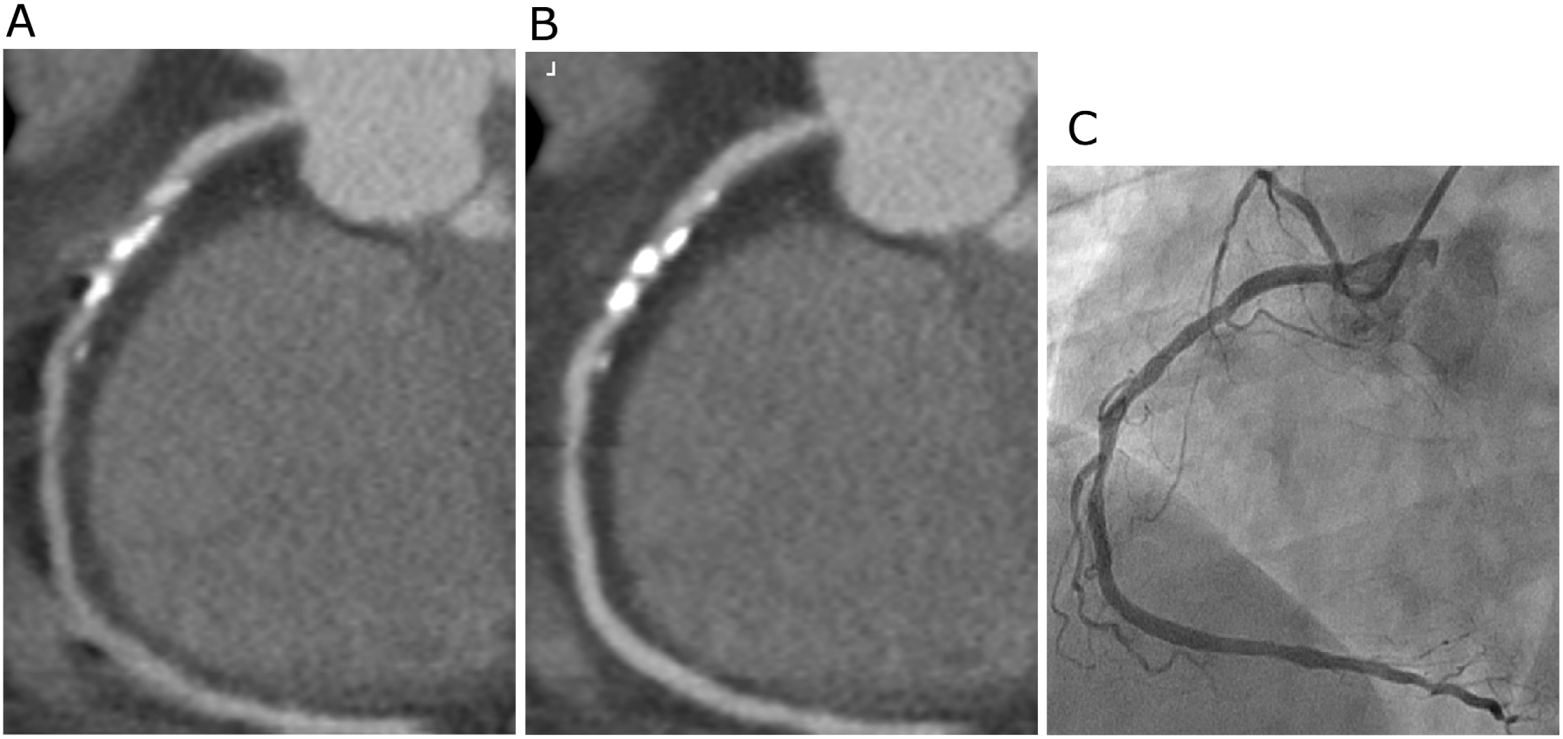
Example of a 57-year-old man (body weight 78 Kg, heart rate 65 beats/min) underwent prospective scan, curved multiplanar reconstruction images of the right coronary artery (RCA) using conventional reconstruction technology (A) and the new motion-correction algorithm (B), both were performed at 75% of the R-R interval, taking invasive coronary angiography image (C) as reference. Motion artifacts observed at the mid RCA (A) were significantly corrected by the new algorithm (B).

A total of 694 coronary segments were analyzed both in motion-correction and conventional reconstructions groups. The motion-correction algorithm significantly improved image quality and interpretability compared with conventional reconstruction method (Table 2). Median image quality score was increased from 3.0 to 4.0 both on overall per-segment level and per-vessel level for left main coronary artery, left anterior descending (LAD) coronary artery, and right coronary artery (RCA) (P<0.001). Inter-observer agreement for image quality was excellent in motion-correction group as well as in conventional reconstruction group, Kappa = 0.864 and Kappa = 0.876 respectively. Overall interpretability was significantly improved, with a significant reduction in the number of non-diagnostic segments (690 of 694, 99.4% vs 659 of 694, 94.9%; P<0.001). However, only the RCA showed statistical difference (45 of 46, 97.8% vs 35 of 46, 76.1%; P = 0.004) on a per-vessel basis in this regard. Improved image quality and interpretability for RCA in one subject was shown in Fig 2.

**Table 2:** Image quality and interpretability between Motion-correction versus Conventional reconstructions.

	Motion-correction reconstructions	Conventional reconstructions	P value
Image quality grade			
LM	4 (3–4)	3 (3–4)	0.001
LAD	4 (3–4)	3 (3–4)	<0.001
LCX	3 (3–4)	3 (2–3)	<0.001
RCA	4 (3–4)	3 (3–4)	<0.001
Overall	4 (3–4)	3 (3–4)	<0.001
Interpretability by artery			
LM	100 (46/46)	97.8 (45/46)	1.000
LAD	100 (46/46)	91.3 (42/46)	0.117
LCX	93.5 (43/46)	86.9 (40/46)	0.485
RCA	97.8 (45/46)	76.1 (35/46)	0.004
Overall Interpretability			
Per-patient	93.5 (43/46)	71.7 (33/46)	0.012
Per-vessel	97.8 (180/184)	88 (162/184)	<0.001
Per-segment	99.4 (690/694)	94.9 (659/694)	<0.001

Values are median (interquartile range) or % (n/N). LAD = left anterior descending coronary artery; LCX = left circumflex coronary artery; LM = left main coronary artery; RCA = right coronary artery.

### Diagnostic performance

**Fig 3:**
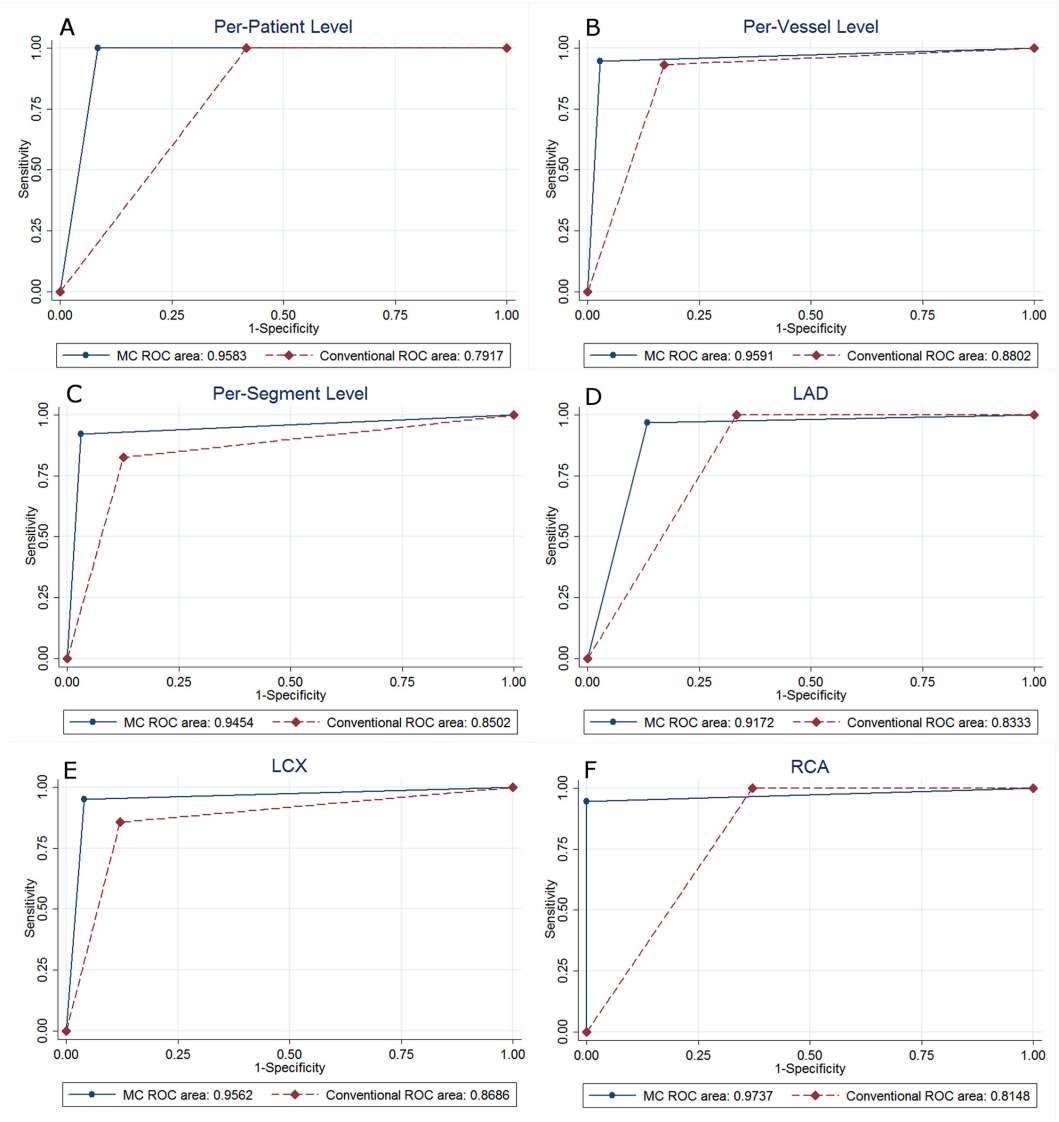
Receiver-operating characteristic (ROC) curves for detection of ≥50% stenosis on per-patient (A), per-vessel (B), per-segment (C) levels, and left anterior descending coronary artery (D), left circumflex coronary artery (E), right coronary artery (F) by artery analysis are shown.

Diagnostic performance for detecting ≥50% stenosis between motion-correction versus conventional reconstructions (Table 3) showed that luminal stenosis ≥50% by ICA was found in 34 of the 46 patients (73.9%), 73 of 184 coronary arteries (39.7%), and 126 of 694 (18.2%) coronary segments. The area under ROC curve analysis showed 0.96 (95% CI: 0.87 to 1.00) and 0.79 (95% CI: 0.65 to 0.94) on per-patient basis, 0.96 (95% CI: 0.92 to 0.99) and 0.88 (95% CI: 0.83 to 0.93) on per-vessel basis, 0.95 (95% CI: 0.92 to 0.97) and 0.85 (95% CI: 0.81 to 0.89) on per-segment basis for motion-correction versus conventional reconstruction groups, respectively, and all of these differences showed statistically significant (Fig 3). Diagnostic accuracy, specificity, and PPV were significantly improved (96.2% vs 87.0%, 97.3% vs 82.9%, 95.8% vs 78.2%, respectively) on the per-vessel level and (96.1% vs 86.6%, 97.0% vs 87.5%, 87.2% vs 59.4%, respectively) on the per-segment level. Sensitivity showed statistically significant difference between two study groups only on the per-segment level. There was no statistically significant difference with regard to NPV between the two study groups on any level. On per-patient level analysis however, there showed no statistically significant difference regarding any of the diagnostic performance characteristics.

**Table 3:** Diagnostic accuracy for detecting ≥50% stenosis by ICA between Motion-correction versus Conventional reconstructions on three levels.

	Motion-correction reconstructions, % (n/N)	Conventional reconstructions, % (n/N)	P value
Per-patient (n = 46)			
Accuracy	97.8 (45/46)	89.1 (41/46)	0.203
Sensitivity	100 (34/34)	100 (34/34)	1.000
Specificity	91.7 (11/12)	58.3 (7/12)	0.155
PPV	97.1 (34/35)	87.2 (34/39)	0.203
NPV	100 (11/11)	100 (7/7)	1.000
AUC (ROC)	0.96 (0.87–1.00)	0.79 (0.65–0.94)	0.019
Per-vessel (n = 184)			
Accuracy	96.2 (177/184)	87.0 (160/184)	0.002
Sensitivity	94.5 (69/73)	93.2 (68/73)	1.000
Specificity	97.3 (108/111)	82.9 (92/111)	<0.001
PPV	95.8 (69/72)	78.2 (68/87)	0.001
NPV	96.4 (108/112)	94.8 (92/97)	0.736
AUC (ROC)	0.96 (0.92–0.99)	0.88 (0.83–0.93)	<0.001
Per-segment (n = 694)			
Accuracy	96.1 (667/694)	86.6 (601/694)	<0.001
Sensitivity	92.1 (116/126)	82.5 (104/126)	0.036
Specificity	97.0 (551/568)	87.5 (497/568)	<0.001
PPV	87.2 (116/133)	59.4 (104/175)	<0.001
NPV	97.0 (551/568)	95.8 (497/519)	0.328
AUC (ROC)	0.95 (0.92–0.97)	0.85 (0.81–0.89)	<0.001

Values are % (n/N). NPV = negative predictive value; PPV = positive predictive value; AUC (ROC) = Area under the ROC

We also investigated the effect of the novel motion-correction algorithm on diagnostic performance by every main coronary artery analysis (Table 4). Only two left main coronary arteries of the 46 patients were detected with ≥50% stenosis by ICA, the other 44 left main coronary arteries were classified as negative for < 50% or no stenosis both by ICA and CCTA, and there was no significant difference for diagnostic performance between the two study groups. The area under the ROC curve analysis showed significant improved diagnostic performance in the motion-correction group both by left circumflex (LCX) coronary artery and RCA analysis, with area under the ROC curve being 0.96 (95% CI: 0.90 to 1.00) versus 0.87 (95% CI:0.77 to 0.97) and 0.97 (95% CI:0.92 to 1.00) versus 0.81 (95% CI:0.72 to 0.91), respectively (Fig 3). There was no statistically significant difference regarding any of the diagnostic performance characteristics between the two study groups both in LAD coronary artery and LCX coronary artery analysis. However, diagnostic accuracy, specificity, and PPV were significantly improved in the RCA analysis (97.8% vs 78.3%, 100% vs 63.0%, 100% vs 65.5%, *p* = 0.007, 0.001, 0.008, respectively).

**Table 4:** Diagnostic accuracy for detecting ≥50% stenosis by ICA between Motion-correction versus Conventional reconstructions on three vessels.

	Motion-correction reconstructions, % (n/N)	Conventional reconstructions, % (n/N)	P value
LAD (n = 46)			
Accuracy	93.5 (43/46)	89.1 (41/46)	0.714
Sensitivity	96.8 (30/31)	100 (31/31)	1.000
Specificity	86.7 (13/15)	66.7 (10/15)	0.390
PPV	93.8 (30/32)	86.1 (31/36)	0.434
NPV	92.9 (13/14)	100 (10/10)	1.000
AUC (ROC)	0.92 (0.82–1.00)	0.83 (0.71–0.96)	0.133
LCX (n = 46)			
Accuracy	95.7 (44/46)	87.0 (40/46)	0.267
Sensitivity	95.2 (20/21)	85.7 (18/21)	0.606
Specificity	96.0 (24/25)	88.0 (22/25)	0.609
PPV	95.2 (20/21)	85.7 (18/21)	0.606
NPV	96.0 (24/25)	88.0 (22/25)	0.609
AUC (ROC)	0.96 (0.90–1.00)	0.87 (0.77–0.97)	0.041
RCA (n = 46)			
Accuracy	97.8 (45/46)	78.3 (36/46)	0.007
Sensitivity	94.7 (18/19)	100 (19/19)	1.000
Specificity	100 (27/27)	63.0 (17/27)	0.001
PPV	100 (18/18)	65.5 (19/29)	0.008
NPV	96.4 (27/28)	100 (17/17)	1.000
AUC (ROC)	0.97 (0.92–1.00)	0.81 (0.72–0.91)	0.003

Values are % (n/N). NPV = negative predictive value; PPV = positive predictive value; AUC (ROC) = Area under the ROC

## Discussion

Three main limitations of CCTA reported include motion artifacts due to rapid or irregular heart rhythm, blooming artifacts from coronary artery calcium or stent, and high radiation dose [3]. Recently, an innovative motion-correction algorithm has been developed to address motion artifacts in CCTA with insufficient or absent rate-control medications. Several previous studies have indicated the algorithm’s beneficial effect on image quality and diagnostic performance [8–15]. However, most of these studies were performed with retrospective ECG gating, with higher overall radiation dose exposures. Prospective ECG triggering CCTA has emerged as an alternative to retrospective ECG gating for coronary artery disease evaluation, and this new method significantly decreases radiation dose while maintaining diagnostic image quality [4,16–18]. This protocol requires strict heart rate control to prevent motion artifacts according to most recommendations. In our analysis, we evaluated the impact of a motion-correction algorithm on image quality and diagnostic performance in prospective ECG triggering CCTA without rate control.

In the present study, 46 subjects were prospectively enrolled with mean heart rate 68.8±8.4 beats/min without rate-control agents. All successfully underwent CCTA using prospective ECG triggering keeping beam time as narrow as possible to reduce radiation dose but also allow sufficient phases for the motion-correction algorithm. Mean radiation dose in our study was 3.3±0.6 mSv, significantly lower than retrospective ECG gating protocol previously reported [16–18]. Our study indicates that the new motion-correction algorithm provides real promise to improve the diagnostic accuracy of prospective ECG triggering CCTA without rate control, being consistent with previous reports by Fuchs and Andreini [9,15].

In our results, we demonstrated that use of a motion-correction algorithm significantly increased median image quality score both on overall per-segment level and per-vessel level for each of the major coronary arteries, suggesting a beneficial effect on image quality of the motion-correction algorithm. The motion-correction algorithm improved interpretability on every analysis level, especially for RCA in the interpretability by artery analysis, which coincides with previous reports by Leipsic and Fuchs [8,9]. We conclude that introduction of the new motion-correction algorithm has certainly improved image quality and interpretability for patients undergoing low-dose CCTA, and the effect of this algorithm is more evident for RCA where motion artifacts mostly occurred.

Image quality and diagnostic performance could potentially be perfect using traditional multi-segment reconstruction in patients with relatively low heart rate, but we are more interested in testing effect of this new reconstruction method in relatively high heart rate populations. Accordingly, we enrolled patients who did not take rate-control drugs before CCTA, and all successfully underwent prospective ECG triggering CCTA. Compared with previous studies performed in retrospective ECG gating [8,10], we got similar results that the new algorithm significantly improved every analysis level, especially on the per-vessel and per-segment levels, diagnostic accuracy, specificity, and PPV. The PPV, rather than NPV, showed statistically significant improvements, and these were further confirmed in our study for prospective ECG triggering CCTA protocol with a relatively larger sample size. CCTA has long been perceived as a powerful diagnostic tool for detection and exclusion of CAD due to its impressively high sensitivity and excellent NPV, which could not be further improved by the new motion-correction technology according to our results as well as previous reports. Notwithstanding, the novel algorithm significantly improved specificity and PPV, which have long been considered less robust in CCTA, suggesting a favorable effect of application of this new algorithm in clinical routine.

Furthermore, we investigated effect of the novel algorithm on diagnostic performance by three main coronary artery analysis, finding that diagnostic performance for RCA including diagnostic accuracy, specificity, PPV were significantly improved by the new algorithm. Mild improvement was observed for LCX artery with the area under the ROC mildly improved. However, there was no significant improvement for LAD artery. Previous knowledge on coronary artery motion shows that [19–21] the greatest amount of coronary motion occurred in RCA, followed in descending order by the LCX and LAD artery, accordingly. Motion artifacts mostly occurred in RCA, where the most evident effect of the motion-correction algorithm was observed.

### Limitations of the study

There were several limitations in the present study. First, similarly to most studies, we recruited patients who had been referred for ICA, which unavoidably introduces bias. Although the prevalence of obstructive disease in our cohort was higher than in comparable investigations, it is still not reflective of the disease prevalence in patients undergoing clinically indicated CTA according to current guidelines. Larger multicenter studies including populations with natural prevalence of obstructive CAD would be desirable to further validate our results. Second, heart rate and heart rate variability have a great impact on the occurrence of motion artifacts. We did not analyze the impact of heart rate and heart rate variability on effect of the new motion-correction algorithm, but larger sample size studies with low and high heart rate subgroup analysis merit further study. As reported by Lee and Andreini [10,15], the new algorithm can significantly improve image quality and interpretability, especially in patients with a higher heart rate. We anticipate similar results in studies with low-dose CCTA. Third, patients with relatively high heart rate (70–90 beats/min for example) usually successfully underwent CCTA on a second-generation dual-source CT scanner without rate control in our clinical routine. We did not investigate if CCTA using single-source CT scanner with the new motion-correction algorithm is comparable to that of CCTA on dual-source CT scanner, and thus necessitates further investigation. The upcoming multicenter clinical trial with a larger sample size may confirm the effect of this new algorithm on CCTA. Further information will include the upper heart rate threshold of utility of the algorithm, and the additive value of the algorithm to traditionally reconstructed CCTA [22]. Fourth, in our study, we aimed to investigate image quality and diagnostic accuracy, rather than to investigate radiation dose. A possible limitation of our study may be not to lowering the radiation dose as low as reasonably achievable. Further studies may investigate more into this concern.

In conclusion, the innovative motion-correction algorithm could be successfully used in prospective ECG triggering CCTA without rate control. Image quality, interpretability, and diagnostic performance could be significantly improved by the new algorithm, particularly for the right coronary artery, in comparison to conventional multi-segment reconstruction method.
